# Insulin receptor alternative splicing in breast and prostate cancer

**DOI:** 10.1186/s12935-024-03252-1

**Published:** 2024-02-08

**Authors:** Jinyu Li, Gena Huang

**Affiliations:** Department of Medical Oncology, The Second Hospital of Dalian Medical University, No. 467 Zhongshan Road, Shahekou District, Dalian, 116023 Liaoning China

**Keywords:** Insulin receptor isoforms, Alternative splicing, Cancer

## Abstract

Cancer etiology represents an intricate, multifactorial orchestration where metabolically associated insulin-like growth factors (IGFs) and insulin foster cellular proliferation and growth throughout tumorigenesis. The insulin receptor (IR) exhibits two splice variants arising from alternative mRNA processing, namely IR-A, and IR-B, with remarkable distribution and biological effects disparities. This insightful review elucidates the structural intricacies, widespread distribution, and functional significance of IR-A and IR-B. Additionally, it explores the regulatory mechanisms governing alternative splicing processes, intricate signal transduction pathways, and the intricate association linking IR-A and IR-B splicing variants to breast and prostate cancer tumorigenesis. Breast cancer and prostate cancer are the most common malignant tumors with the highest incidence rates among women and men, respectively. These findings provide a promising theoretical framework for advancing preventive strategies, diagnostic modalities, and therapeutic interventions targeting breast and prostate cancer.

## Introduction

The human insulin receptor (IR) gene consists of 22 exons, and exon 11 undergoes selective splicing to form two IR isoforms, IR-A and IR-B [1]. Multiple splicing factors regulate the process of selective splicing of the IR. The two isoforms generated have different distribution patterns in various tissues and organs of the human body. They exhibit significant differences in their ligand-binding affinity for insulin and insulin-like growth factors, as well as in the signal transduction pathways they induce and the biological effects they exert. These differences not only contribute to essential factors such as insulin resistance and type 2 diabetes but also impact the growth, proliferation, and apoptosis of tumor cells. As the most common malignant tumors with the highest incidence rates among men and women, prostate cancer and breast cancer have been experiencing a continuous increase in their incidence rates. In 2023, there were 1,958,310 newly diagnosed cancer cases in the United States, averaging over 5370 cases daily. Among women, breast cancer, lung cancer, and colorectal cancer they accounted for 52% of all new cancer cases, with breast cancer comprising 31% of female cancers. Among men, prostate cancer, lung and bronchus cancer, and colorectal cancer accounted for 48% of all new cancer cases, with prostate cancer comprising 29% of male cancers [2]. This review summarizes the regulation of IR selective splicing, tissue-specific distribution, and signal transduction under physiological and pathological conditions. Additionally, we discuss the relevance of the splicing isoforms IR-A and IR-B to breast and prostate cancer. Future research on the different IR isoforms and their signaling pathway molecules may provide new diagnostic and therapeutic targets for the clinical treatment of breast and prostate tumors, thereby improving the survival outcomes of cancer patients.

## The structure, distribution, and function of IR isoforms

**Fig. 1:**
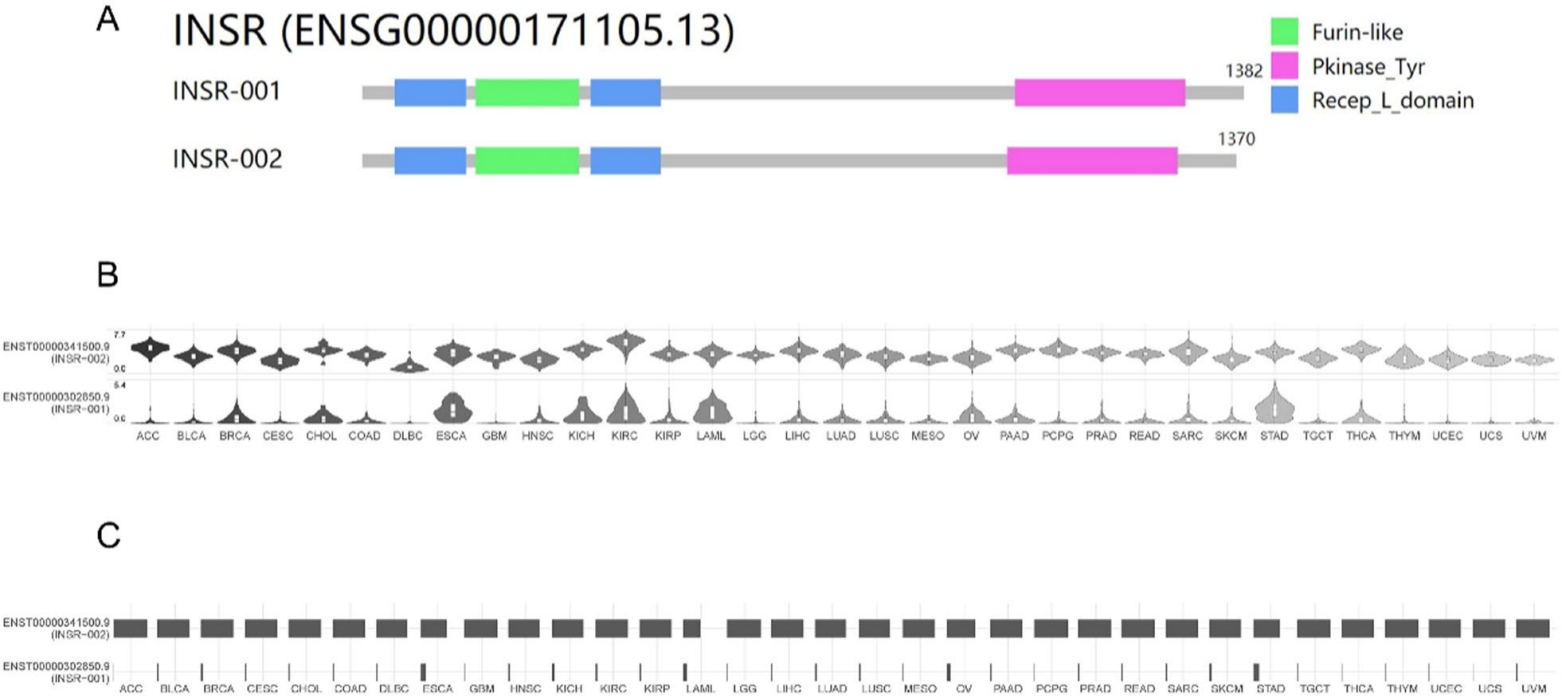
Expression of IR-A and IR-B in different cancer types. **A** IR isoforms in GEPIA (Gene Expression Profiling Interactive Analysis). INSR-001: IR-B, INSR-002: IR-A. Furin-like: The furin-like structure domain is capable of cleaving specific peptide chains, thus participating in the processing and activation of certain proteins during the alternative splicing process. Pkinase_Tyr: The Pkinase_Tyr domain is capable of regulating the activation status of relevant signaling pathways during alternative splicing. Recep_L_domain: The Recep_L domain structure can mediate RNA-RNA or RNA–protein interactions during the process of alternative splicing, thereby influencing splice site selection and efficiency. B.C Violin plot (**B**) and box plot (**C**) showing the expression level of IR-A and IR-B in different cancer types by isoform usage profiling in GEPIA. *INSR-001* IR-B, *INSR-002* IR-A. *ACC* Adrenocortical carcinoma, *BLCA* Bladder Urothelial Carcinoma, *BRCA* Breast invasive carcinoma, *CESC* Cervical squamous cell carcinoma and endocervical adenocarcinoma, *CHOL* Cholangiocarcinoma, *COAD* Colon adenocarcinoma, *DLBC* Lymphoid Neoplasm Diffuse Large B-cell Lymphoma, *ESCA* Esophageal carcinoma, *GBM* Glioblastoma multiforme, *HNSC* Head and Neck squamous cell carcinoma, *KICH* Kidney Chromophobe, *KIRC* Kidney renal clear cell carcinoma, *KIRP* Kidney renal papillary cell carcinoma, *LAML* Acute Myeloid Leukemia, *LGG* Brain Lower Grade Glioma, *LIHC* Liver hepatocellular carcinoma, *LUAD* Lung adenocarcinoma, *LUSC* Lung squamous cell carcinoma, *MESO* Mesothelioma, *OV* Ovarian serous cystadenocarcinoma, *PAAD* Pancreatic adenocarcinoma, *PCPG* Pheochromocytoma and Paraganglioma, *PRAD* Prostate adenocarcinoma, *READ* Rectum adenocarcinoma, *SARC* Sarcoma, *SKCM* Skin Cutaneous Melanoma, *STAD* Stomach adenocarcinoma, *TGCT* Testicular Germ Cell Tumors, *THCA* Thyroid carcinoma, *THYM* Thymoma, *UCEC* Uterine Corpus Endometrial Carcinoma, *UCS* Uterine Carcinosarcoma, *UVM* Uveal Melanoma

The IR gene is located on chromosome 19 and consists of 22 exons and 21 introns [3, 4]. The IR protein is a heterotetramer consisting of two α subunits located in the extracellular region and two β subunits spanning the cell membrane. Both the α and β subunits are encoded by the mRNA transcribed from the 22 exons of the IR gene. The mRNA derived from the IR gene produces a protein with a length of 1370 amino acids, approximately equivalent to 154 kDa in size (Fig. 1A). After translation and post-translational modifications, furin protease enzymatically cleaves it, resulting in the production of two distinct subunits: an α subunit consisting of 723 amino acids with an approximate mass of 130 kDa and a β subunit comprising 620 amino acids weighing around 95 kDa. The extracellular domain of the IR includes the complete α subunit chain along with a segment of the β subunit chain containing 194 amino acid residues. In comparison, the intracellular region consists only of the remaining β subunit chain (containing 403 amino acid residues), which includes the tyrosine kinase activity domain. Ligands of the IR bind to the extracellular portion of the α subunit, activating the tyrosine kinase domain of the intracellular β subunit. Once activated, it triggers the phosphorylation of the β subunit, initiating a chain reaction of signaling pathways that elicit various biological effects [5–10].

Exon 11 encodes a unique sequence of 12 amino acid residues that appear towards the C-terminus of the IR α-subunit. During the transcription process, exon 11 undergoes selective splicing, forming two distinct isoforms of the receptor: IR-A and IR-B. Various research articles have extensively studied the structure, distribution, and function of IR isoforms [10–14]. IR-A lacks exon 11, whereas IR-B includes it. These isoforms exhibit structural, distributional, and functional disparities. IR-A displays predominant upregulation in tumor tissues, the brain, hematopoietic stem cells, and embryonic tissues. In contrast, IR-B exhibits strong expression primarily in insulin-responsive target organs such as the liver, adipose tissue, and skeletal muscle. This differential expression underlies the distinct roles played by IR-A and IR-B, with IR-B being mainly involved in metabolic-related effects [11, 14, 15]. Proteins and pathways primarily associated with insulin-activated IR-A were involved in cancer, stemness, and interferon signaling. Instead, proteomic analysis mostly involved IR-B-expressing cells in metabolic or tumor-suppressive functions [16]. Due to the lack of the 12 amino acid residues encoded by exon 11 in the alpha subunit of IR-A, the interaction between the ligand insulin and IR-A is more dynamic, facilitating binding and dissociation processes [17–19]. IR-A demonstrates a greater affinity towards IGF-2, IGF-1, and proinsulin when compared to IR-B [20, 21]. Irregular or abnormal expression of different isoforms of the IR has been detected in cancerous cells, leading to increased responsiveness to insulin and insulin-like growth factor II [22]. The abnormal expression identified may be involved in promoting cancer by amplifying the effects of elevated insulin levels, as commonly seen in obese individuals and type 2 diabetic patients.

Additionally, due to their high degree of homology, both isoforms of the insulin receptor, IR-A and IR-B, can form heterodimers with IGF-1R subunits comprising alpha and beta subunits. This interaction leads to the formation of hybrid receptors, specifically Hybrid-Rs, which consist of Hybrid-RsA and Hybrid-RsB [23]. According to reports, Hybrid-RsB explicitly exhibits a high affinity towards IGF-1, whereas Hybrid-RsA exhibits pronounced affinities for IGF-1, IGF-2, and insulin [24]. Additionally, studies have indicated that both Hybrid-RsB and Hybrid-RsA exhibit lower affinity for insulin in hamster ovarian cells and neonatal kidney cells, while their affinities are disproportionately higher for IGF-1 and IGF-2 [25, 26]. Consequently, variations in the distribution of IR splice variants under physiological and pathological conditions result in the activation of distinct signaling pathways and consequential biological effects influenced by insulin and IGFs.

## Regulating alternative splicing of the IR

**Fig. 2:**
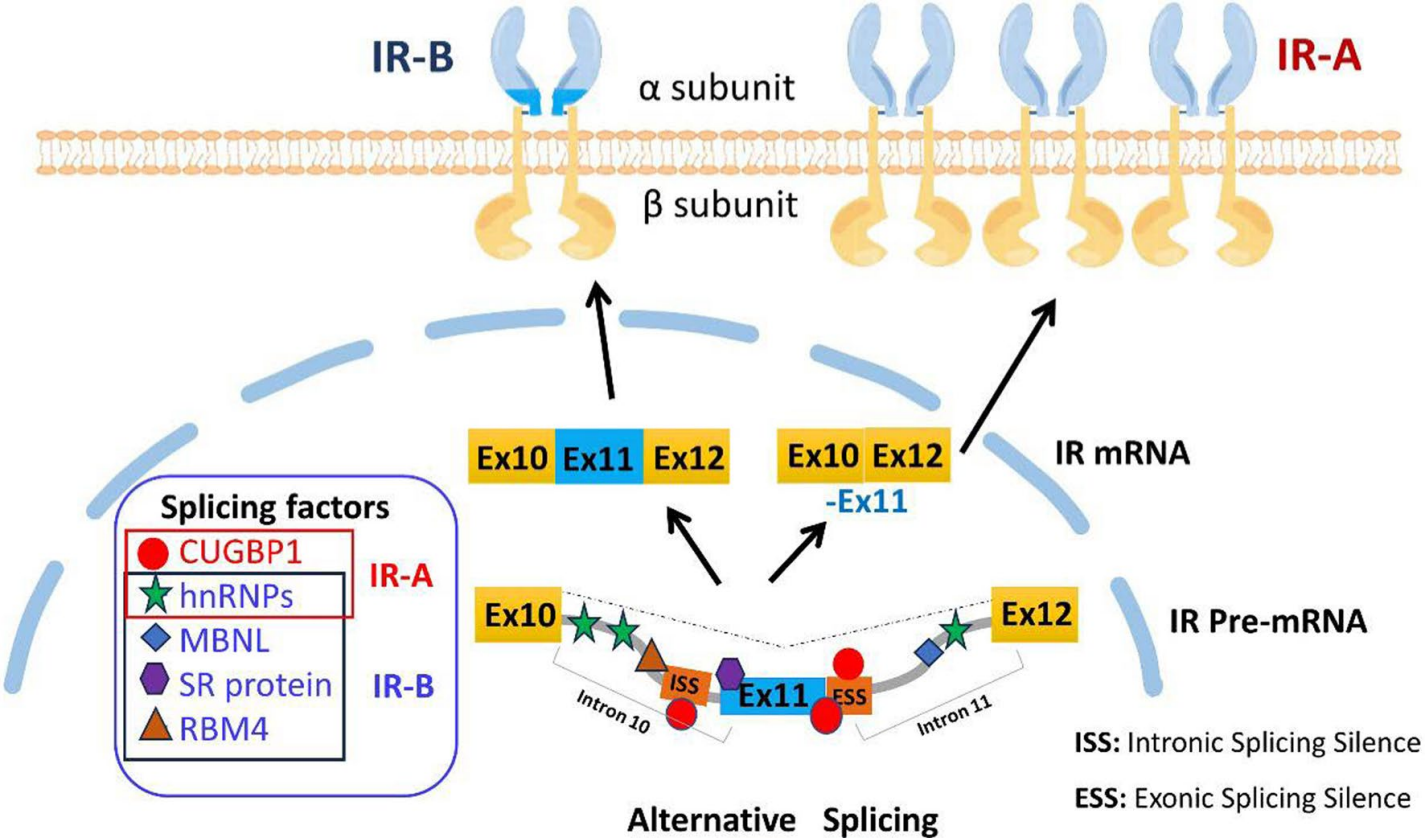
Proposed model for the regulation of IR pre-mRNA alternative splicing by splicing factors in cancers. The diagram provides an overview of the primary regulators that influence insulin IR activity at both the promoter and mRNA levels. Splicing factors play a crucial role in controlling IR gene transcription by either promoting or inhibiting it. Multiple splicing factors are involved in regulating IR expression after transcription. Once the IR mRNA is formed, splicing factors remove introns and facilitate the joining of exons. They also regulate the alternative splicing of exon 11, resulting in the production of either IR-A (exon11-) or IR-B (exon11 +) isoforms. In cancer cells, there is an elevated expression of the IR-A isoform, which can have oncogenic effects. *ISS* Intronic Splicing Silence, *ESS* Exonic Splicing Silence

Alternative splicing of the IR is a complex process that involves various factors and mechanisms. Several studies have investigated the regulation of IR alternative splicing and its implications in different physiological and pathological conditions [13, 22, 27–31]. After the IR gene is transcribed, the pre-mRNA undergoes a complex intron removal and exon ligation process facilitated by spliceosomes and multi-component ribonucleoprotein complexes. The exonic and intronic sequences of pre-mRNA contain binding sites for various splice-related RNA-binding proteins, known as splicing factors, and positive/negative regulatory elements. These splicing factors recognize and interact with specific splice sites on pre-mRNA, either promoting or inhibiting the assembly of spliceosomes, thereby finely regulating the splicing process. Therefore, it is essential to identify the specific regulatory sequences and splicing factors that play a role in the targeted splicing of the IR gene. This knowledge is pivotal for understanding alternative splicing regulation mechanisms and determining the relative proportion of spliced isoforms IR-A to IR-B. Notably, the sequences within intron 10 and exon 11 contain several positive/negative regulatory motifs that play significant roles in the context of selective splicing of the IR [32]. Splicing factors intricately control whether exon 11 is incorporated or omitted during mRNA processing, influencing the tissue-specific expression patterns observed for IR-A and IR-B spliced isoforms (Fig. 2). These highly regulated processes exhibit specificity during development and at different time points, resulting in tissue-specific variations in the relative abundance and distinct biological properties displayed by these isoforms [14]. The proportion between IR-A and IR-B indirectly indicates the distribution and expression levels of specific splicing factors governing this regulatory mechanism. Noteworthy examples of these splicing factors include CELFs (CUG-binding protein), members of the Elav-like family, hnRNPs (heterogeneous nuclear ribonucleoproteins), MBNLs (Muscle blind-like proteins), SR proteins (serine-arginine-rich), and RBM4 (RNA-binding motif protein 4). Their interplay contributes to the precise control of alternative splicing events associated with the IR gene.

The involvement of the CELFs protein family is vital in governing multiple facets of mRNA processing, encompassing alternative splicing, editing, and translation regulation. Among the members of this family, CUG-binding protein 1 (CUGBP1) was the first identified splicing factor involved in the selective splicing of the IR [33]. CUGBP1 selectively attaches to two silencer sequences: one found prior to exon 11 and at the 3ʹ end of intron 10, and the other located precisely on exon 11. Both silencer elements contribute to the promotion of exon 11 splicing, thereby facilitating the expression of the spliced isoform IR-A. This intricate mechanism highlights the significant role of CUGBP1 and its impact on the regulation of IR splicing events[34, 35]. hnRNP proteins influence the intricately regulated processes of mRNA, encompassing alternative splicing, translation control, and ensuring stability [36]. Talukdar et al. [37] have reported the involvement of two hnRNPs, namely hnRNP F and hnRNP A1, in the selective splicing of the IR. These proteins bind to splice regulatory elements rich in GA sequences within introns and exons. Specifically, hnRNP F selectively binds to the termini of intron 10, enhancing the inclusion of exon 11 and facilitating the expression of the spliced isoform IR-B. Conversely, hnRNP A1 targets the 5ʹ end of both intron 10 and intron 11, exerting an opposing impact to hnRNP F. By promoting exon 11 splicing, hnRNP A1 drives the expression of the spliced isoform IR-A. Muscle blind-like protein 1 (MBNL1) recognizes and binds to a highly conserved enhancer element on intron 11, which facilitates the expression of IR-B [34]. Ho et al. [38] also found that Muscleblind proteins regulate alternative splicing of the IR. Additionally, MBNL1 can counteract the splicing function of CUGBP1 and engage with other splicing factors participating in the alternative splicing of INSR mRNA. For example, it hinders the splicing function of hnRNP H, leading to increased expression of the IR-B isoform. [39]. The SR protein family plays a critical role in mRNA alternative splicing by binding to splice sites on exons or introns and interacting with small nuclear ribonucleoproteins (snRNPs) [40]. SRp20 and SF2/ASF, among other splicing factors, attach themselves to the enhancer sequence located at the 5ʹ end of exon 11, leading to an increased expression of IR-B. According to Sen et al. [34], these splicing factors can oppose the splicing function of CUGBP1 and engage with other splicing factors, consequently modifying the IR-A/IR-B ratio. Similarly, RBM4 regulates mRNA alternative splicing and translation by binding to sequences rich in GC content, facilitating the inclusion of exon 11 and enhancing the expression of IR-B. Lin et al. noticed an increase in IR-A expression in both embryonic fibroblasts and muscle tissues of RBM4 gene knockout mice [41]. In hepatic carcinomas, IR-A is overexpressed due to EGFR-mediated dysregulation of RNA splicing factors by upregulating the expression of the splicing factors CUGBP1, hnRNPH, hnRNPA1, hnRNPA2B1, and SF2/ASF [42]. Nakura et al. [43] reported that Rbfox, acting as a splicing regulator, is involved in exon 11 splicing. Huang et al. [35]. discovered that the splicing factor CUGBP1 plays a role in controlling the balance between IR-A and IR-B in breast cancer cells and impacts tumor cell biological responses through the IR signaling pathway.

## The signaling pathways of IR isoforms

**Fig. 3:**
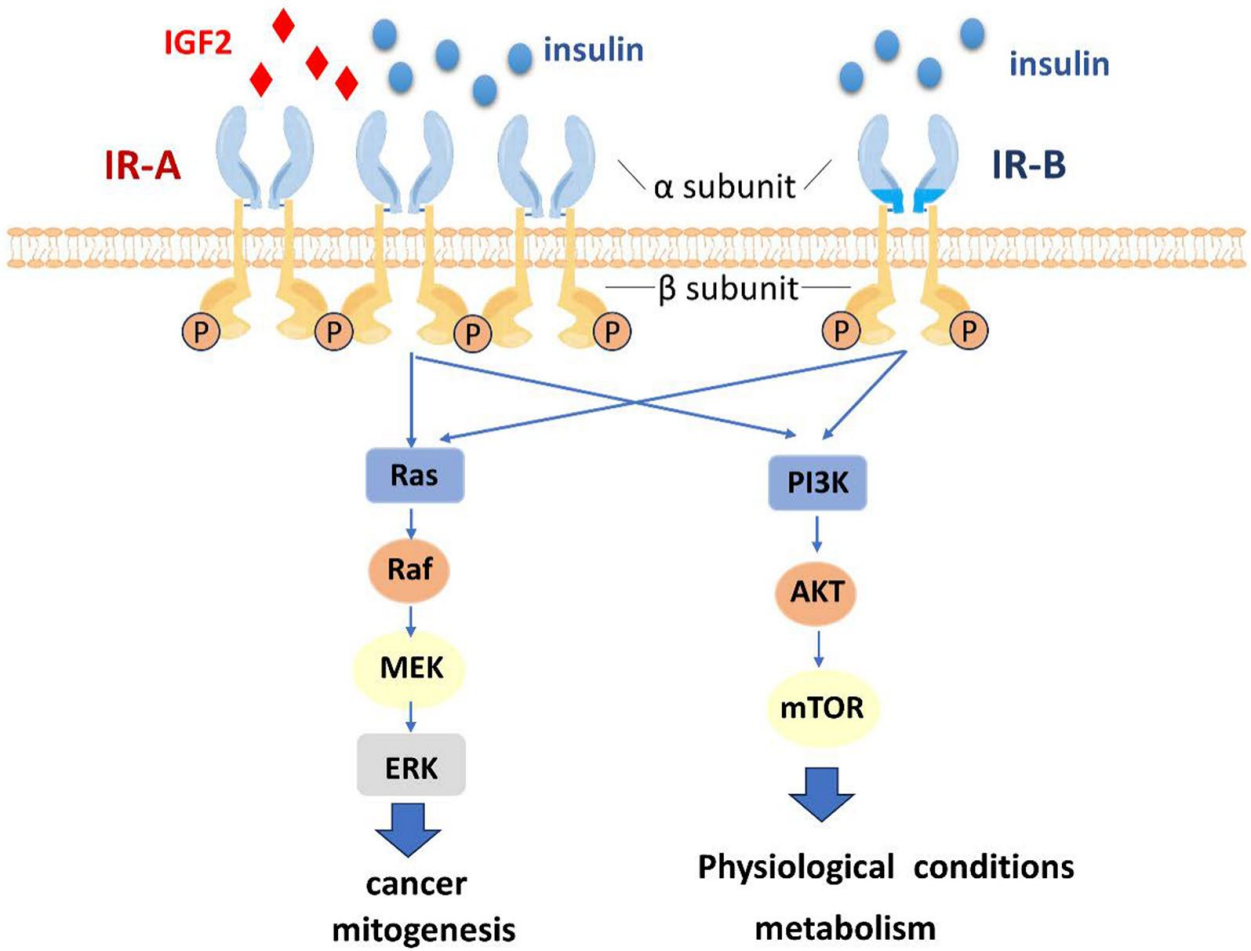
Proposed model for the IR signal diversification and partitioning in cancers. Activation of IR isoforms leads to the activation of specific signaling pathways, such as the PI3K-Akt pathway and the MAPK pathway, which regulate various cellular processes.In cells where IR-A is primarily expressed and IGF-2 is produced, such as fetal or cancer cells, activation of IR-A by IGF-2 promotes non-metabolic responses, including cell proliferation and movement. On the other hand, in cells and tissues with a higher expression of IR-B, insulin activation of IR-B supports metabolic and physiological functions through downstream signaling

Insulin, an essential hormone, plays a critical role by binding to and activating IR, influencing various cellular functions through diverse signaling pathways [44]. Insulin binding to the extracellular α subunit of IR triggers structural modifications resulting in autophosphorylation of the intracellular β subunit of IR. Consequently, the activated IR tyrosine kinase phosphorylates multiple substrates within the cell, including IR substrates (IRS) and Src homology 2 domain-containing protein family (Shc), both serving as adaptor proteins for downstream signaling [45, 46]. IRS proteins harbor numerous tyrosine phosphorylation sites that act as binding regions for adaptor proteins containing the Shc domain. These adaptor proteins further recognize and activate tyrosine phosphorylation residues, such as phosphoinositide 3-kinase (PI3K) and growth factor receptor-bound protein 2 (Grb-2). Activation of PI3K and Grb-2 mediates metabolic effects and mitogenic effects, respectively [44, 45]. The metabolic effects of glucose in skeletal muscle, adipose tissue, and liver involve the participation of the PI3K/Akt signaling pathway, particularly in processes like glucose absorption, gluconeogenesis, and glycogen synthesis [47, 48]. Additionally, under insulin stimulation, this signaling pathway promotes nitric oxide production in endothelial cells, leading to vasodilation [49]. On the other hand, the Grb-2/P44/42 MAPK signaling pathway exerts regulatory effects on gene transcription, protein synthesis, cell growth, and differentiation. Furthermore, it impacts the secretion of endothelin-1 in endothelial cells [23, 50]. Consequently, due to variations in tissues and different ratios of IR isoforms (IR-A and IR-B), insulin stimulation elicits separate signaling pathways that give rise to different biological responses (Fig. 3).

IGF-2 primarily stimulates IR-A, promoting cell growth and invasion while causing abnormal nuclear localization of IRS-1 in parental 32D hemopoietic cells [51]. However, IGF-2 does not bind to IR-B. Under insulin stimulation, IR-B predominantly mediates cell differentiation and metabolic effects [51]. For instance, in human uterine smooth muscle tumor cells, IGF-2 stimulates IR-A to activate the Grb-2/P44/42 ^mapk^ signaling pathway, thereby promoting cell migration. Conversely, insulin stimulates IR-A to activate the PI3K/Akt signaling pathway, inhibiting apoptosis [52]. IGF-2 prompts IR-A activation in R-/IR-A cells lacking IGF-1R expression while expressing only IR-A. Consequently, IR-A activation triggers Akt/glycogen synthase kinase-3β (Akt/GSK3β) activation, thereby mediating mitogenic effects [53]. Under the same conditions, p70S6 kinase (p70S6K), P44/42 ^mapk^, and Akt can also be activated, indicating that IR-A has complex effects beyond the signaling pathways induced by IGF-2 stimulation [54]. In pancreatic beta-cell lines, differing membrane distributions of IR isoforms allow for differential activation of promoters. The Insulin promoter is activated via the IR-A pathway, while the glucokinase promoter is activated via the IR-B insulin receptor pathway. Consequently, insulin activates the IR-B/PI3K/AKT pathway, enhancing the transcription of the glucose kinase (GK) gene. Simultaneously, the expression level of insulin itself is regulated through the IR-A/PI3K/p70S6K pathway [55].

Another critical signaling pathway activated by IR isoforms is the MAPK pathway. The pathway regulates cellular processes such as cell proliferation, differentiation, and survival [56]. IR isoforms can activate the MAPK pathway by recruiting adaptor proteins and activating downstream kinases [57]. Proinsulin is generally regarded as an inactive prohormone because of its low metabolic activity; in R-/IR-A cells, where IGF-1 demonstrates lower affinity for IR-A compared to IGF-2, the downstream activation of the ERK and Akt signaling pathways remains significant [20]. Moreover, stimulation of IR-A by both IGF-1 and IGF-2 results in higher ratios of downstream signaling molecules, such as p70S6K/Akt and ERK1/2/Akt, when contrasted with insulin stimulation of IR-A [54]. Despite its low metabolic activity, proinsulin is generally considered an inactive prohormone; in cells expressing only IR-A, proinsulin stimulates cell proliferation and migration through IR-A, exhibiting similar effects to IGF-2 and insulin. The intracellular signaling pathway molecules resemble those activated by IGF-2 stimulation, with a higher p70S6K/Akt ratio than insulin stimulation of IR-A. In contrast to insulin, proinsulin exhibits slower negative feedback mechanisms in the activation of IR. The degradation of IR and substrate IRS-1 requires 24 h of stimulation for proinsulin, while insulin only requires 8 h. This difference explains why proinsulin elicits a stronger mitogenic and migratory effect [20]. In conclusion, the functionality of IR isoforms, IR-A and IR-B, is critical in mediating the signaling pathways of insulin. Once activated, these isoforms initiate unique signaling pathways, including the PI3K-Akt pathway and the MAPK pathway, which regulate various cellular processes. Dysregulation of IR isoform signaling is implicated in the development of metabolic disorders and cancer.

## IR isoforms in breast and prostate cancer

In many cancers, the expression of the selective splicing isoform IR-A of the IR is noticeably elevated compared to IR-B (Fig. 1B, C), particularly in conditions of compensatory hyperinsulinemia [58, 59]. The increased expression of IR-A and an elevated IR-A: IR-B ratio facilitate the proliferative response of cancer cells to insulin and insulin-like growth factor 2 (IGF-2) [59]. Nowak-Sliwinska et al. show that IR-A is the main splice variant in tumor vasculature, which may impact tumor angiogenesis and angiostatic treatment [60]. Furthermore, extensive research has demonstrated a significant correlation between the expression of IR-A and IR-B with that of IGF1-R [24, 25, 61]. Intriguingly, IR isoforms exhibit a more pivotal role in specific tumor tissues when compared to IGF1-R itself. The varying levels of IR isoform expression across diverse cancer types have potential implications for prognosis and survival. These findings provide valuable insights into the underlying mechanisms and offer new perspectives for targeted therapy against IGF, thereby opening up novel target sites for future therapeutic interventions [62].

Increased expression of IR-A in cancer is influenced by various factors within the intricate regulatory network, including changes in transcription factors and dysregulation of microRNA. Splicing factors, such as CUGBP1, hnRNP proteins, SF2/ASF, and SRp20/SRSF3, have been reported to contribute to the dysregulation of the IR-A: IR-B ratio in cancer. In studies focusing on hepatocellular carcinoma, the IR-A: IR-B ratio was consistently elevated compared to the adjacent non-tumor liver tissue. This increase in ratio was associated with the upregulation of splicing factors, including CUGBP1, hnRNPH, hnRNPA1, hnRNPA2B1, and SF2/ASF [42]. SRp20/SRSF3 have the potential to prevent hepatic carcinogenesis by modulating IGF-2 and IR-A, thereby influencing Wnt/β-catenin signaling, inducing c-Myc, and leading to aberrant splicing and induction of EMT genes [63]. MicroRNAs (miRNAs) exhibit frequent dysregulation in human cancers and play a crucial role as potent oncogenes. MiR-424, in addition to its role in IR regulation, is crucial in inhibiting the growth of cancer cells and is widely recognized as a tumor suppressor in diverse cancer types [64, 65]. In breast cancer [66], miR-195 inhibits tumor angiogenesis by suppressing the IRS1-VEGF axis. MiR-195 is also identified as a tumor suppressor in non-small cell lung cancer (NSCLC) cells, directly targeting the IGF-1R [67]. Furthermore, there may be an interplay between the dysregulation of microRNAs and splicing factors. A specific instance of this can be observed in bladder cancer, where miRNA-1 inhibits the function of the serine/arginine-rich splicing factor 9 (SRSF9/SRp30c) [68].

### IR isoforms in breast cancer

The role of IR isoforms in breast cancer has been the subject of investigation in multiple studies. Vigneri et al. [1] reviewed the role of insulin, IR, and cancer, including breast cancer, and emphasized the activating effects of insulin on cancer cell growth, mainly through the involvement of its specific receptor instead of the IGF-1 receptor. Belfiore et al. [57] explored the involvement of IR isoforms and hybrid insulin/IGF-I receptors in human cancer, explicitly focusing on breast cancer. They drew attention to the potential existence of an autocrine/paracrine growth loop wherein IR-A expression and local abundance of IGF-II in breast tumors play a significant role. In conclusion, this suggests that IR isoforms, particularly IR-A, may promote breast cancer cell growth. In a separate study, Huang et al. [69] investigated the altered expression of IR isoforms in breast cancer. Their findings revealed a higher IR-A/IR-B ratio in breast cancer tissues compared to normal breast tissues.

Multiple studies have consistently demonstrated that the expression levels of IR-A are significantly higher, while those of IR-B are noticeably lower in breast cancer tissues. These findings have been confirmed through diverse methodologies, such as breast cancer cDNA microarrays, qPCR arrays, and analysis of clinical tissue samples [70–72]. Vella et al. found that a higher IR-A/IR-B ratio is associated with shorter disease-free survival in breast cancer and that a higher IR-A is associated with a poorer outcome in human TNBC. They also evaluated the diverse biological role of the two IR isoforms when expressed in murine TNBC cells [73]. Moreover, Harrington et al. [72] identified that ER + breast cancer displays significantly higher levels of IR-A compared to ER- breast cancer, while no notable difference was observed in IR-B expression. Furthermore, in hormone therapy-resistant ER + breast cancer, there is a significant increase in both the expression level of IR and the ratio of IR-A to IR-B. At the same time, there is a significant decrease in IGF1-R expression. Additionally, within ER + breast cancer, the luminal B subtype presented a higher IR-A to IR-B ratio than the luminal A subtype. Huang et al. [35] similarly found that luminal breast cancer cells exhibit considerably heightened levels of IR-A and demonstrate a more excellent IR-A/IR-B ratio when compared to other subtypes of breast cancer cell lines. This observation highlights the potential impact of the IR signaling pathway on tumor cell biological responses. A large-scale clinical cohort study revealed a correlation between phosphorylated IR/IGF-1R (pIGF-1R/IR) levels and unfavorable prognosis. It is worth noting that antibodies used in the study could not differentiate between p-IR and p-IGF-1R; downstream signaling molecule p-S6K (phospho-S6) levels were found to correlate with IR expression rather than IGF-1R expression. These observations suggest that the phosphorylation of IR, coupled with its downstream signaling pathways, contributes to influencing the prognosis of individuals diagnosed with breast cancer [74]. Furthermore, the IR isoforms have been implicated in metabolic reprogramming in cancer cells. The IR-A isoform, along with its ligand IGF2, can modulate the metabolic pathways in breast cancer cells [75]. It suggests that IR-A may have a role in cancer metabolic reprogramming, contributing to cancer progression and metastasis. These collective findings shed light on the crucial role of exploring IR isoforms in breast cancer research, providing potential insights for prognostic assessment and guiding targeted therapeutic interventions.

### IR isoforms in prostate cancer

Prostate cancer is a complex disease that is influenced by various signaling pathways, including those involving IR isoforms. Sciacca et al. [48] conducted a study on insulin analogs and their differential activation of IR isoforms in three engineered cell models (IGF1R (-), IGF1R-deprived mouse fibroblasts transfected with either only human IR-A or IR-B or IGF1R), the results of their investigation suggest that long-acting analogs stimulate the mitogenic signaling pathway with greater efficacy than insulin, leading to heightened cell proliferation. These observations suggest that IR isoforms, specifically IR-A, potentially contribute to mediating the mitogenic effects exerted by insulin in prostate cancer cells. Furthermore, Vella et al. [59] provided a comprehensive review of IR isoforms in cancer, including prostate cancer. They illustrated that the aberrant expression of IR isoforms may contribute to the growth and progression of prostate cancer.

Furthermore, variations in the expression of IR isoforms have been noted in prostate cancer [76]. In their meticulous investigation, Cox et al. thoroughly assessed IR and IGF-1R expression levels in tissue samples obtained from patients with prostate cancer, comparing them to samples collected from normal prostate tissues. Their findings revealed a significant upregulation of IR expression in prostate cancer tissues compared to normal prostate tissues. Using qRT-PCR, they quantified the IR-A/IR-B ratio and found a marked elevation of this ratio in prostate cancer tissues compared to neighboring non-cancerous tissues and normal prostate tissues [77]. In parallel research, Heidegger et al. unveiled the impact of insulin and IGF-1 on cell behavior in prostate cancer cell lines. They demonstrated that insulin and IGF-1 promoted cell proliferation and heightened glucose metabolism in these cells. Conversely, in normal prostate cells, insulin and IGF-1 induced cellular differentiation. Notably, overexpression of IR-A and IGF-1R facilitated cell proliferation in tumor cells and stimulated cell differentiation in normal tissue cells. However, overexpression of IR-B did not contribute to tumor cell proliferation [78]. Further validation using in vivo models confirmed that overexpressing IR-A and IGF-1R promoted tumor cell growth, induced angiogenesis, and generated drug resistance. While overexpression of IR-B also induced angiogenesis to some extent, its potency was notably weaker than that of IR-A and IGF-1R [79]. Moreover, Perks et al. demonstrated a consistent pattern by showing significantly higher IR-A expression than IR-B in both prostate cancer tissues and prostate cancer cell lines. Additionally, stimulation with insulin and IGF-2 led to an upregulation of IR-A expression [80]. Collectively, these groundbreaking findings provide valuable insights into the intricate function of the IR signaling pathway in the context of prostate cancer. The upregulated expression of IR-A in prostate cancer tissues suggests its potential as a therapeutic target. At the same time, the observed effects on cell proliferation, differentiation, angiogenesis, and drug resistance shed light on the complex interplay between insulin signaling and tumor biology. Further investigation into these mechanisms may open new avenues for precision medicine approaches in prostate cancer treatment.

## Targeting IR selective splicing variants: potential for clinical therapeutics

The pursuit of inhibitors that target IR selective splicing variants has emerged as a promising avenue for clinical therapeutics. These inhibitors hold the potential to modulate the expression and functionality of distinct insulin isoforms, presenting a customized approach to precision medicine in the management of diverse diseases.

Several studies have provided evidence to support that the insulin/IGF-2/IR-A pathway and its downstream signaling cascades serve as specific and distinct target sites in malignant tumors. The interplay between IR and IGF1-R, forming heterodimeric receptors, significantly impacts malignancies [81]. In tumor cells with an autocrine loop of IGF-2, targeting IGF1-R leads to compensatory upregulation of phosphorylated IR [82]. Knockout of IGF1-R in mouse embryonic fibroblasts enhances IR signaling, while inhibiting IR enhances IGF-1R signaling [83, 84]. Furthermore, overexpression of IR-A in cancer cells confers resistance to monoclonal antibody therapy, such as trastuzumab (an anti-IGF1-R antibody), suggesting that IR expression can serve as a predictive molecular marker for resistance to tumor-targeted therapies [85]. Therefore, in the context of malignant tumors, targeting the insulin/IGF-2/IR-A pathway primarily involves these approaches: dual intervention against both IR and IGF-1R using small molecule tyrosine kinase inhibitors (TKIs), specific targeting of IR-A function, and suppression of the ligand IGF-2, which activates both IR and IGF-1R, IR-A isoform-specific aptamers, nucleotides oligomers and selective splice-switching antisense oligonucleotides. The implications of these findings indicate the promise of directing therapeutic interventions toward the insulin/IGF-2/IR-A pathway in the context of malignant tumors. By modulating this pathway, it may be possible to disrupt tumor cell growth, overcome resistance to targeted therapies, and improve patient outcomes. Further study is needed to assess the efficacy and safety of implementing these approaches in a clinical environment, paving the way for novel therapeutics tailored to the unique molecular characteristics of malignant tumors.

Linsitinib (OSI-906) and BMS-754807 are well-established dual-targeting small molecule tyrosine kinase inhibitors (TKIs) specifically designed to block IR and IGF-1R selectively. Preclinical studies have shown that Linsitinib effectively inhibits the activity of both IR and IGF-1R in tumor cells and tumor xenograft models. Tumors with an autocrine loop of IGF-2 and high phosphorylation levels of IR and IGF-1R exhibit high sensitivity to Linsitinib [86]. Linsitinib therapy reverses tamoxifen resistance caused by activated IGF-1R in ER-positive breast cancer [87]. A single-arm phase II study investigated the use of Linsitinib in mCRPC [88], and the study found that single-agent Linsitinib was safe and well tolerated. However, further research is needed to identify the specific population that may benefit from this treatment. Similar results to Linsitinib have been observed using BMS-754807, either as a single therapy or alongside other chemotherapy medications like gefitinib, gemcitabine, and cisplatin [89, 90]. The combination of BMS-754807 with other small molecule inhibitors or radiotherapy may represent a rational therapeutic approach in prostate cancer [91, 92]. Zanella et al. reported that the level of IGF-2 expression can predict the sensitivity of tumors to EGFR-targeted therapy, and the sensitivity to combined IGF-2/EGFR targeted therapy is closely related to the level of IGF-2 [93]. The primary function of MEDI-573, an IgG2 monoclonal antibody developed from humans, is to neutralize IGF-1 and IGF-2, inhibiting their ability to activate IGF-1R and IR-A [94]. In a mouse model, overexpression of IGF-2 leads to colorectal cancer, and the application of MEDI-573 effectively reduces the level of IGF-2 expression and inhibits the growth of colorectal tumor cells [95]. The domain 11 of M6P/IGF2R exhibits strong binding capability to IGF-2, leading to a decrease in its expression level in serum and subsequently inhibiting the biological effects of IGF-2. Mutations in the binding site of domain 11 enhance the binding capacity to IGF-2 by approximately 100-fold, significantly reducing the level of serum IGF-2 [96]. In light of preclinical studies that demonstrate higher IR-A/IR-B ratios among individuals with hormone receptor positive, ERBB2 negative breast cancer, a phase I/II clinical trial is presently underway to explore the potential impact of MEDI-573 in conjunction with hormonal therapy within this particular subset of breast cancer patients [97, 98]. IR-A and IR-B undergo maturation in the Golgi apparatus through cleavage by furin protease. When furin loses its activity, IR-A and IR-B are translocated to the cell surface, with IR-B matured through the catalysis of the convertase PACE4. Thus, furin protease plays a critical role in facilitating the maturation of IR-A. Polyphenols (catechins, ellagic acid, and quercetin) can inhibit furin protease; thereby, the maturation of IR-A is decreased, leading to a reduction in its downstream signaling and cellular mitotic activity [99–101].

Nucleic acid-based aptamers, molecules derived from nucleic acids, are showing potential as therapeutic agents that can counteract disease-associated proteins like receptor tyrosine kinases. A recent study has described a nuclease-resistant RNA aptamer that can specifically recognize and inhibit the IR, thereby blocking IR-dependent signaling pathways. The findings suggest that it may be feasible to identify aptamers with high affinity that specifically bind to the IR-A isoform, providing a targeted approach for modulating its activity [102]. Several specific miRNAs, including miR-424, miR-195, miR-497, miR-103/107, and miR-1, play a regulatory role in controlling the expression of IR under physiological and pathological conditions such as obesity and insulin resistance [103]. These miRNAs show altered expression patterns in cancer, potentially contributing to increased levels of IR and an elevated IR-A: IR-B ratio.

## Conclusion

The IR undergoes intricate regulatory processes during selective splicing, resulting in two distinct isoforms, IR-A and IR-B, which exhibit divergent distribution patterns and functionalities under both physiological and pathological conditions. The variation in the ratio between these isoforms plays a crucial role in pathological conditions like cancer and diabetes. Despite their minimal structural differences, the absence of antibodies capable of distinguishing between IR-A and IR-B poses a challenge in studying their biological characteristics. Consequently, investigations have predominantly relied on assessing mRNA expression levels or transfecting cells with exogenously overexpressed isoforms. To comprehensively comprehend the unique functions and responsibilities played by these isoforms in different physiological and pathological contexts, it is essential to conduct in vivo studies using diverse disease models, organs, and tissues. Future research should focus on regulating all factors involved in the expression and mRNA splicing of the IR gene and the protein processing of the two IR isoforms. Additionally, it is essential to recognize that the complexity of IR signaling diversification arises from multiple isoforms, each with unique characteristics, including varying affinities for ligand binding, distinct membrane organization and movement, and the ability to interact with a diverse array of molecular partners. Consequently, these isoforms can selectively impact downstream signaling pathways based on their attributes. Such research endeavors are vital in establishing a robust theoretical foundation for future advancements in clinical precision medicine. By unraveling the complexities of IR isoforms, we can pave the way for targeted therapies tailored to individual patients, revolutionizing treatment approaches and improving outcomes.

## Data Availability

No data was used for the research described in the article.
